# Psychometric evaluation of a new proxy‐instrument to assess participation in children aged 3–6 years with visual impairment: PAI‐CY 3‐6

**DOI:** 10.1111/opo.12642

**Published:** 2019-08-29

**Authors:** Ellen B M Elsman, Ruth M A van Nispen, Gerardus H M B van Rens

**Affiliations:** ^1^ Department of Ophthalmology Amsterdam Public Health research Institute Amsterdam UMC Vrije Universiteit Amsterdam Amsterdam The Netherlands; ^2^ Department of Ophthalmology Elkerliek Hospital Helmond The Netherlands

**Keywords:** children, item response theory, low vision, participation, questionnaire, validation

## Abstract

**Purpose:**

The Participation and Activity Inventory for Children and Youth 3–6 years (PAI‐CY 3‐6) was recently developed to assess the participation needs of children with visual impairment (VI) by means of parent‐proxy report. This study reports on its psychometric properties.

**Methods:**

Parents of children aged 3–6 years registered at two low vision rehabilitation centers in the Netherlands were invited to participate and completed the 52‐item PAI‐CY. Satisfaction with the PAI‐CY 3‐6 was determined using an evaluation form. Basic item analyses was conducted, after which an item response theory (IRT) model (i.e. the graded response model, GRM) was fitted. Deletion of items was informed by results of item analyses, fulfillment of IRT assumptions, differential item functioning, fit to the GRM and item information content. Face and content validity were considered; professionals from low vision rehabilitation centers were asked for their opinion in the item deletion process. After obtaining a satisfactory set of items, known‐group validity, concurrent validity and test‐retest reliability were also investigated.

**Results:**

Data of 237 parents were included in the analyses. Various aspects of the PAI‐CY 3‐6 were perceived as neutral to positive by over 85% of the respondents. After removing 17 items, the remaining 35 items reflected satisfactory fit to the GRM. Known‐group validity was supported, since participants with more severe VI and comorbidity scored significantly worse than those with less severe VI and without comorbidity after correcting for potential confounders. Test‐retest reliability was adequate, and the PAI‐CY showed to have good concurrent validity. Feedback from professionals motivated the maintenance of 3 of the 17 deleted items, although not included in the scoring. Furthermore, two new items were added, resulting in a 40‐item instrument.

**Conclusions:**

The PAI‐CY 3‐6 has sound psychometric properties and can now be used to assess the participation needs of children aged 3–6 years with VI by means of proxy. Implementation in routine low vision rehabilitation care enables further optimization and investigation of its acceptability and feasibility.

## Introduction

Although the prevalence of childhood visual impairment (VI) is low,^1^ it has lifelong and profound implications for both the child and its family, influencing development, education, and physical, social and psychological well‐being.^2^, ^3^, ^4^, ^5^, ^6^ As a result, the needs of children with VI and their parents should be assessed from a life‐time perspective. Recently, an increasing emphasis has been placed on patient‐based assessment of the impact of diseases on functioning, participation, and quality of life. This has led to the development of generic and disease specific patient‐reported outcome measures (PROMs).^7^, ^8^, ^9^, ^10^, ^11^, ^12^

After a paucity, several instruments for pediatric ophthalmology have been developed in recent years, measuring either vision‐related quality of life or functional vision. Most instruments use self‐reports of children with VI, targeting children >5 years.^13^, ^14^, ^15^, ^16^, ^17^, ^18^ The Children's Visual Function Questionnaire (CVFQ) and the Pediatric Eye Questionnaires (PedEyeQ) are currently the only instruments for use in young children, using parent‐proxy reports. The CVFQ consists of two versions, one for children <3 years, and one for children ≥3 years.^19^ Although the name suggests differently, it was developed to measure vision‐related quality of life and it assesses competence, personality, family impact and treatment difficulty imposed by specific eye conditions, rather than overall visual function. For young children, the PedEyeQ has parent‐proxy versions for 0–4 and 5–11 years, measuring functional vision and eye‐related quality of life.^20^ This instrument is not specifically targeted at children with VI, but is aimed at the evaluation of eye‐related concerns. As such, no instrument is currently available to measure developmentally‐appropriate participation specifically for children with VI.^21^

The Participation and Activity Inventory for Children and Youth (PAI‐CY) has recently been developed in the Netherlands, to assess the participation needs of children with VI and their parents.^2^, ^22^ Since needs change with increasing age and development,^2^, ^23^ four age‐appropriate versions of the PAI‐CY were created, according to the age‐categories of the World Health Organization (WHO): 0–2, 3–6, 7–12 and 13–17 years. The PAI‐CY is going to be used for diagnostic purposes at the Dutch low vision rehabilitation centers, who currently use a semi‐structured approach, resulting in underrepresentation of needs,^24^, ^25^ hampering referral to rehabilitation programs and possibly the quality of care provided.^26^ The content of the PAI‐CY was driven by the most important stakeholders (i.e. parents of children with VI, children with VI aged 7–17 years and professionals working at low vision rehabilitation centers) using online questionnaires and concept‐mapping workshops,^2^ strengthening its content validity. Feasibility and acceptability of the PAI‐CY were tested in a pilot study.^22^ In the current study, the psychometric properties of the PAI‐CY 3‐6 were assessed in order to improve its content for use in future research and practice.

## Methods

The study protocol was approved by the Medical Ethical Committee of the Amsterdam UMC, Amsterdam, the Netherlands. This study was performed in accordance with the ethical standards as laid down in the Declaration of Helsinki. Written informed consent was obtained from all included participants.

### Participants

Parents/caretakers (parents for brevity) of children aged 3–6 years with VI registered at two Dutch low vision rehabilitation organizations (Royal Dutch Visio and Bartiméus) were invited to participate (*n* ≈ 1180). Parents had to have adequate knowledge and understanding of the Dutch language to fill in the questionnaires. Parents of children with VI from any cause were eligible, and there was no restriction regarding visual performance. Children with profound cognitive impairment which was registered in the patient files at the low vision rehabilitation organizations were excluded from the selection to be invited by the low vision rehabilitation organizations, because the questions would concern activities not applicable to them because of the developmental delay. Children with mild cognitive impairment, which was not registered in the patient files but reported by parents, could participate.

### Procedures

Parents who agreed to participate were asked to fill in questions regarding sociodemographic and clinical characteristics of their child, the PAI‐CY 3‐6, a self‐constructed evaluation form, and the Dutch version of the Child and Adolescent Scale of Participation (CASP).^27^ Ophthalmic diagnoses, decimal visual acuity, and visual field of children were retrieved from the patient files at the low vision rehabilitation centers. Missing values in patient files were complemented with self‐reported data from parents (*n* = 37 for visual performance, *n* = 46 for diagnoses). Decimal visual acuity was transformed into logMAR and put into 5 levels based on the better seeing eye, according to the WHO criteria for VI:^28^ logMAR ≤ 0.3 (≥20/40) referred to ‘no VI’, logMAR 0.31–0.52 (20/40–20/66) to ‘mild VI’, logMAR 0.53–1 (20/66–20/200) to ‘moderate VI’, logMAR 1.01–1.30 (20/200–20/400) to ‘severe VI’ and logMAR ≥ 1.31 (≤20/400) to ‘blind’. A visual field ≤ 10 degrees was classified as ‘blind’;^28^ otherwise only visual acuity was used for classification. Ophthalmic diagnoses were combined to describe the site of VI (i.e. whole globe and anterior segment, glaucoma, cornea, lens, uvea, retina, optic nerve, cerebral/visual pathways, other and unknown). A retest on the PAI‐CY 3‐6 was conducted after 2 weeks, which is the recommended time interval, as children have probably remained stable (i.e. the same answers are expected), but parents are unlikely to have remembered their answers because of the length of the PAI‐CY 3‐6.^29^ By default, parents filled in the questionnaires through a web‐based survey questionnaire, but if requested, they could also receive a paper‐and‐pencil version (*n* = 3).

The preliminary version of the PAI‐CY 3‐6 comprises 52 items grouped into 12 domains (for descriptive purposes only, in order to provide contextual meaning) that were informed by the concept‐mapping workshops with end‐users:^2^ attachment (AT‐5 items), incentive processing (IP‐4 items), visual attention (VA‐4 items), orientation (OR‐3 items), mobility (MO‐6 items), play (PL‐3 items), social relationships (SR‐6 items), motor functioning (MF‐2 items), communication (CO‐4 items), school/daycare (SD‐6 items), reading and writing (RW‐5 items), and self‐reliance (SE‐4 items). Each item is scored on a 4‐point Likert scale with response options: not difficult (1), slightly difficult (2), very difficult (3), and impossible (4). The response option ‘not applicable’ is treated as a missing value. After each domain a question is asked to clarify rehabilitation needs. In addition to the 52 items, there are 10 items regarding sensory functioning and 8 items regarding parental experiences. These items are not considered to be part of the construct measured by the other 52 items of the PAI‐CY 3‐6, and were therefore outside the scope of this study.

The Dutch version of the CASP was used as comparator instrument to assess concurrent validity. The CASP measures children's extent of participation and restrictions in home, school and community life situations and activities compared with same‐age peers as reported by a parent or caregiver.^27^, ^30^, ^31^ The CASP was selected because, at the time of this study, it was evaluated most extensively, generally showing moderate positive results.^21^, ^32^, ^33^ It has been used in children from 3 years of age, and the Dutch version showed to have good measurement properties among a population of children with acquired brain injury.^27^, ^30^

### Statistical analyses

Prior to conducting item response theory (IRT) analyses, some basic item analyses were performed and IRT assumptions were checked.

#### Initial item analyses

First, participants with >25% missing responses on the PAI‐CY 3‐6 were removed from the analyses. The best performing items were selected using an iterative process. Moreover, evaluation forms and comments of parents were also considered, as was the researchers’ expertise. Furthermore, four professionals from Dutch low vision rehabilitation centres who conduct the diagnostic assessment procedure and are the intended end‐users of the PAI‐CY 3‐6 were asked for their opinion about item maintenance or removal.

Items with >70% of the respondents endorsing the first or last answer category (floor or ceiling effect) were considered for deletion, as were items not having an answer in one of the response categories. Items with missing scores 20%–40% were considered for deletion as well, while items with missing scores >40% were deleted immediately from further analyses. Items showing inter‐item correlations >0.7, indicating similarity and potential redundancy, were also considered for deletion.

#### IRT assumptions

Principal component analysis (PCA) was used to assess the unidimensionality assumption. By calculating the acceleration factor, indicating points of abrupt change in the scree plot, the number of factors was assessed.^34^ To verify that all items load on one component, magnitude of principal components were checked. Possible covariation (>0.25) among items in the residual PCA matrix was inspected to assess local independence. Item pairs with excess covariation were considered for deletion; the least performing item was selected. Monotonicity was evaluated using Mokken scale analyses, and the resulting graphs were visually inspected. A Loevinger H coefficient was calculated to assess scalability;^35^, ^36^, ^37^ a value <0.3 was considered unsatisfactory.

#### Calibration using the graded response model

One of the most common IRT models for questionnaires with ordinal responses, the graded response model (GRM), was used to estimate discrimination (α) and threshold parameters (β).^38^, ^39^ Using a likelihood ratio test (LRT), a full model was compared with a constrained model^40^, ^41^ which was nested within the full model and has equal discrimination parameters (similar to the Rasch model).^42^ The usability of the IRT model depends upon how well the model accurately reflects the data. Therefore, model fit and individual item fit were investigated. Indices to assess overall fit of the selected model were the root mean square error of approximation (RMSEA), standardized root mean square residual (SRMR), comparative fit index (CFI), and Tucker‐Lewis index (TLI).^43^ The CFI and TLI should be around 0.95 or higher, whereas the SRMR should be around 0.08 or lower and the RMSEA around 0.06 or lower.^44^ Individual item fit was assessed using the X^2^ statistic, with significant results indicating misfit.^45^, ^46^ To assess functioning of items, the information content of items in relation to the total test information (i.e. item information) was inspected. Information reflects how precisely an item measures the underlying trait, and as such represents reliability or measurement precision.^40^, ^47^ The Item Information Curves (IICs) show the amount of information an item holds along the underlying trait, and at which point at the underlying trait individuals can best be discriminated by an item.^40^, ^48^ Information is usually highest in the area where the threshold parameters are located, and highly discriminating items normally contribute more information.^47^ Items with low information across the latent trait were considered for deletion, but IICs, Category Response Curves (CRCs) and content validity were also taken into account.^47^ If items covered the same range on the latent trait, the item with least information and/or holding information over the narrowest range was considered for deletion.

#### Differential item functioning

After selecting the best performing items, a person‐item map was computed to evaluate whether item difficulty matches ability of participants.^49^ Differential item functioning (DIF) was inspected to assess whether participants with different characteristics and the same disability level have equal probabilities of selecting a certain item response.^29^, ^47^ DIF is uniform if an item is endorsed either more or less at all values of the latent trait by one of the groups, whereas DIF is non‐uniform if it occurs not equally at all values of the latent trait.^29^ Using an iterative hybrid of logistic regression and IRT, the Likelihood Ratio χ^2^ test at α level 0.01 was used as detection criterion, and McFadden's pseudo *R* ^2^ was used as a measure for the DIF magnitude, with a 2% change being considered as critical value.^50^ DIF was evaluated for age (median split: <5 vs ≥5 years), gender (male vs female), and level of VI (no VI/mild VI vs moderate VI/severe VI/blind).

#### Known‐group validity, concurrent validity, test‐retest reliability

To reassure the PAI‐CY 3‐6 was able to differentiate between groups, known‐group validity was investigated for the following groups:^29^ severity of VI (no VI/mild VI vs moderate VI/severe VI/blind), sex, age (3–4 vs 5–6 years), presence of comorbidity including cognitive impairment, parents’ nationality (Dutch vs other), parents’ financial situation (usually enough money vs just enough money/not enough money), and parents’ years of education. Thetas of relevant groups were compared using independent samples t‐tests. Significant differences between groups were at least expected for severity of VI, although differences between groups regarding presence of comorbidity, level of education and financial situation might also be significant. Multiple linear regression including all variables was performed to correct for confounding. Concurrent validity, showing the relationship between summary scores of the PAI‐CY 3‐6 and summary scores of the CASP, was assessed by the Spearman correlation.^29^ A negative correlation >0.4 was expected between scores of the PAI‐CY 3‐6 and the CASP. Test‐retest reliability represents the extent to which responses of participants who have not changed are the same over time.^29^ Test‐retest reliability of the PAI‐CY 3‐6 was assessed using weighted kappa and percentage agreement.^29^ Kappa values >0.4 were considered moderate, >0.6 good and >0.8 very good.^51^ Agreement of 60%–74% was considered moderate, 75%–89% good and ≥90% excellent.^52^ Furthermore, differences in GRM parameters were between test and retest were investigated.

All statistical analyses related to IRT were conducted in R.^53^ The remaining analyses were performed using SPSS version 22.^54^

## Results

### Patient characteristics

Parents of 284 children (response rate ~24%) provided informed consent to participate in the study, of whom 256 (90.1%) completed the first PAI‐CY 3‐6. Data from 19 participants were excluded from the analyses because of too many missing responses (*n* = 17) or because children were already 7 years (*n* = 2; inclusion of these respondents did not affect GRM parameters). Sociodemographic and clinical characteristics of the included participants are presented in *Table* *1*. The retest was completed by 218 parents after a mean of 33.7 ± 29.5 (range 11–164, median 21) days.

**Table 1:** Sociodemographic and clinical characteristics of participants (*n* = 237)

Age in years, mean ± S.D. (range)	4.6 ± 1.0 (3–6)
Male gender, *n* (%)	140 (59.1)
Site of VI, *n* (%)	
Whole globe and anterior segment	7 (3.0)
Glaucoma – primary or secondary	3 (1.3)
Cornea (sclerocornea and corneal opacities)	4 (1.7)
Lens (cataract and aphakia)	16 (6.8)
Uvea	3 (1.3)
Retina	62 (26.2)
Optic nerve	8 (3.4)
Cerebral/visual pathways	49 (20.7)
Other (idiopathic nystagmus, high refractive error)	68 (28.7)
Unknown	17 (7.2)
Severity of VI, *n* (%)^28^	
No VI: logMAR ≤ 0.3 (≥20/40)	86 (36.3)
Mild VI: logMAR 0.31–0.52 (20/40–20/66)	47 (19.8)
Moderate VI: logMAR 0.53–1.00 (20/66–20/200)	72 (30.4)
Severe VI: logMAR 1.01–1.30 (20/200–20/400)	10 (4.2)
Blind: logMAR ≥ 1.31 (≤20/400) or visual field ≤ 10 degrees	6 (2.5)
Unknown	16 (6.8)
Comorbidity, *n* (%)	98 (41.4)
Cognitive impairment, *n* (%)	47 (19.8)
Parent who completed the questionnaire, *n* (%)	
Mother	189 (79.7)
Father	24 (10.1)
Mother and father together	20 (8.4)
Caretaker	4 (1.7)
Nationality parent, *n* (%)	
Dutch	223 (95.3)
Other	11 (4.7)
Education in years parent, mean ± S.D. (range)	13.1 ± 2.6 (0–16)
Financial situation parent, *n* (%)	
Usually enough money	139 (59.4)
Just enough money	43 (18.4)
Not enough money	6 (2.6)
No answer	46 (19.7)

### Initial item analyses

Distribution of responses over the response categories of all 52 items is presented in *Table* *2*. Two items (RW4 and RW5) had over 40% missing values and were deleted immediately, whereas three items had missing values over 20%. Seven items showed floor effects. Response categories 3 (‘very difficult’) and 4 (‘impossible’) were collapsed for all items because of infrequent endorsement of the fourth category, which would reverberate the fit of the IRT model. Eleven item pairs showed high inter‐item correlations. The 50 remaining items comprised a unidimensional scale, mostly yielding high factor loadings. Twenty item pairs showed local dependence and all items fulfilled the monotonicity assumption, although one item had an H coefficient <0.3 (PL3). Because the violations were not very severe, items that did not fulfill the criteria were still included in the first iteration of IRT.

**Table 2:** Distribution of responses over the response categories, and reason for deletion (italic items are maintained, but not included in the scoring)

Item	Item content†	Missing responses (%)	Response distribution by response categories				Phase* and reason for deletion
			1	2	3	4	
AT1	Recognizing facial expressions^a^	0.4	52.5	32.2	13.6	1.7	
AT2	Imitating facial expressions^a^	2.1	56.5	28.0	11.6	3.9	1: Low information; high inter‐item correlation with AT1; local dependence with AT1
AT3	*Recognizing faces of familiar people*	1.3	72.2	22.7	4.3	0.9	1: Floor effect; low information; local dependence with AT1
AT4	Imitating actions or behavior	1.3	67.5	23.9	6.0	2.6	
AT5	Exploring the environment independently	0.4	50.4	41.5	5.9	2.1	
IP1	Reacting to visual stimuli	5.5	75.5	21.4	2.7	0.5	
IP2	Reacting to (sudden) sounds	1.3	44.9	40.2	14.1	0.9	
IP3	Recognizing familiar sounds	0.8	88.5	8.1	2.6	0.9	1: Floor effect; low information
IP4	Executing tasks	0.8	64.7	25.1	7.7	2.6	
VA1	Looking at a particular item	1.7	55.4	35.6	7.3	1.7	1: Low information; local dependence with VA2, VA3 and VA4
VA2	Looking at something for a longer time	2.1	38.4	38.8	20.7	2.2	
VA3	*Following a toy or person with the eyes*	1.7	55.8	31.8	11.2	1.3	1: Low information; local dependence with VA1, VA2 and VA4
VA4	Alternating visual attention	1.7	62.7	27.9	8.2	1.3	
OR1	Orienting in a room	0.0	40.9	47.3	10.1	1.7	
OR2	Exploring the environment by touch	7.2	75.9	21.8	2.3	0.0	
OR3	Finding toys in a closet or toy box	0.4	37.7	44.5	16.1	1.7	
MO1	Being free in movement	0.4	60.6	32.2	5.9	1.3	3: Local dependence MF2; item misfit
MO2	Cycling	6.8	24.4	48.0	19.5	8.1	
MO3	Participating in traffic	16.9	10.2	38.1	29.4	22.3	
MO4	Participating in physical activity classes^b^	11.8	30.6	52.6	11.5	5.3	
MO5	Playing outside	0.4	48.3	40.3	10.2	1.3	
MO6	*Sporting* ^b^	24.1	21.7	52.8	17.2	8.3	2: Missing responses; high inter‐item correlation with MO4; local dependence with MO4
PL1	Playing imaginable games^c^	3.4	69.4	15.7	8.7	6.1	
PL2	Manipulating toys	0.8	62.6	30.2	6.8	0.4	
PL3	Entertaining alone	0.4	58.9	27.5	12.7	0.9	
SR1	Making contact with other children	0.0	61.2	27.9	9.7	1.3	
SR2	Playing outside with friends^d,e^	3.0	50.0	31.7	12.6	5.7	2: High inter‐item correlation with SR3 and SR6
SR3	Playing with children without a visual impairment^d^	2.5	60.2	27.3	10.8	1.7	
SR4	Participating in group activities	13.1	27.7	40.3	25.2	6.8	
SR5	Keeping up with other children while playing	1.7	25.3	40.3	25.3	9.0	
SR6	Participating at others’ birthday parties^e^	19.4	48.7	28.3	17.3	5.8	4: Missing responses, feedback professionals (similar to SR4)
MF1	Cutting and pasting	1.3	34.6	34.6	25.2	5.6	
MF2	Climbing and clambering	1.7	45.1	39.5	11.2	4.3	
CO1	Expressing in words properly^f, g, h^	0.4	61.4	23.7	11.0	3.8	1: High inter‐item correlation with CO2, CO3 and CO4; local dependence with CO2, CO3 and CO4
CO2	Sharing experiences^f, i^	0.8	60.4	21.7	11.5	6.4	
CO3	Asking questions^c, g, I, j^	0.4	65.7	19.5	8.5	6.4	1: High inter‐item correlation with PL1, CO1, CO2 and CO4; local dependence with CO1, CO2 and CO4
CO4	Asking for help^h, j^	0.4	58.9	28.8	8.9	3.4	
SD1	Finding the way in school	9.7	72.9	21.0	3.3	2.8	
SD2	Maintaining overview in the classroom	5.5	30.8	49.1	17.9	2.2	
SD3	Recognizing colors	2.5	66.2	19.5	9.5	4.8	3: Low information; item misfit; bad‐looking category response curve
SD4	Getting insight in concepts	3.0	59.1	27.4	7.8	5.7	
SD5	Maintaining enough energy after school for fun activities	4.6	41.2	38.5	18.1	2.2	
SD6	Finding the coat back independently	3.4	72.9	19.7	4.4	3.1	4: Floor effect, feedback professionals (too specific)
RW1	Recognizing pictures	2.5	51.1	38.1	6.9	3.9	
RW2	Interest in letters^k^	17.3	62.2	18.9	10.7	8.2	1: Low information; high inter‐item correlation with RW3; local dependence with SD4 and RW3
RW3	Recognizing letters^k^	22.4	47.8	27.2	16.3	8.7	
RW4	Initial reading	45.2	45.4	20.0	20.0	14.6	0: Missing responses
RW5	Initial writing	42.2	32.9	30.7	21.2	15.3	0: Missing responses
SE1	Drinking independently	0.4	81.8	11.9	4.7	1.0	
SE2	Eating independently	0.8	67.7	24.7	6.0	1.7	
SE3	(Un)dressing independently	1.3	44.4	29.1	19.7	6.8	
SE4	Tying shoelaces independently	34.6	14.8	20.0	30.3	34.8	2: Missing responses, feedback professionals (too specific)

Note

^a, b, c, d, e, f, g, h, i, j, k^: similar superscript letters indicate item pairs with high inter‐item correlation (>0.7).

AT, attachment; CO, communication; IP, incentive processing; MF, motor functioning; MO, mobility; OR, orientation; PL, play; RW, reading and writing; SD, school/daycare; SE, self‐reliance; SR, social relationships; VA, visual attention.

*Phases: 0: prior to IRT analyses; 1: first iteration of IRT analyses; 2: second iteration of IRT analyses; 3: third iteration of IRT analyses; 4: fourth iteration of IRT analyses.

^†^Not an official (forward‐backward) translation.

The last column in *Table* *2* presents the reason for item deletion. In total, four iterations of item analyses and IRT motivated the deletion of 17 items, resulting in a final item set of 35 items. Besides the statistical reasons for item removal, content validity, similarity to other items and feedback from professionals working at low vision rehabilitation centers and participants was also taken into account. For example, it was decided to remove AT2 instead of AT1, because AT1 was considered to be more important for content validity. Additionally, professionals suggested to delete CO2 and maintain CO1, but this led to too much violations in assumptions (local dependence and high inter‐item correlations). Based on their feedback, it was also decided to maintain three items (AT3, VA3 and MO6), but to not include them in (future) scoring.

### IRT assumptions

Of the remaining 35 items, five items displayed floor effects (IP1, OR2, SD1, and SE1). None of the items was redundant according to cut‐off criteria for inter‐item correlations. As suggested by the acceleration factor, the items were part of a unidimensional scale; principal components were all positive and acceptable. A two factor solution yielded no substantial explained variance: the first factor explained 36% of the variance, while the second factor explained 7%. The ratio of 5.1 between the first and second factor is higher than the required minimum of 4.^55^ From these results, it was concluded that the items comprised a unidimensional scale. Two out of the 595 possible item pairs violated the local independence assumption (MO2‐MO3 and SR1‐SR3). It was decided not to remove one of the items because the violation was not very severe (0.269 and 0.267 respectively) and both items of the pairs were considered to be important for content and face validity. One item violated the monotonicity assumption (MF2), whereas none of the items had a Loevinger H coefficient <0.3.

### Differential item functioning

**Figure 1:**
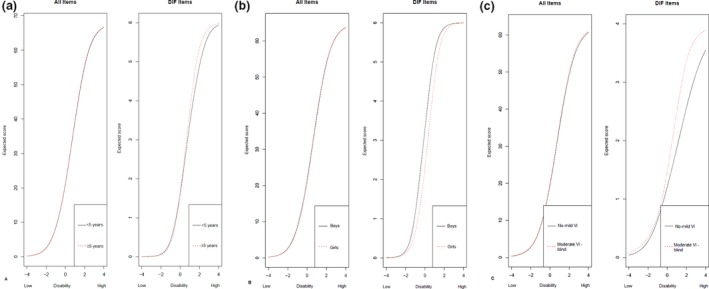
Total impact of DIF on the test characteristic curve (TCC) for age (a), gender (b), and level of VI (c).

*Table* *3* shows McFadden's pseudo R^2^ and IRT parameters for items displaying DIF. Three items (VA4, SR3 and SE3) showed uniform DIF for age, but change in McFadden's pseudo *R* ^2^ was less than 2% for SR3. For items VA4 and SR3, parents of older children were more likely to endorse higher response categories (signaling more difficulty) than parents of younger children with similar disability, whereas for item SE3 parents of younger children endorsed higher response categories. Analyses of DIF for gender indicated three items with uniform DIF (SR4, MF1, and SE3), although change in McFadden's pseudo *R* ^2^ was less than 2% for SE3. For all items, parents of boys were more likely to endorse higher response categories than parents of girls with similar disability. One item (MF2) showed non‐uniform DIF for level of VI, and one item (AT1) showed uniform DIF, with parents of children with more severe VI (moderate‐blind) being more likely to endorse higher response categories compared to parents of children with less severe VI (no‐mild) with similar disability. *Figure* *1* shows the total impact of DIF on the test characteristic curves (TCCs), displaying the relation between the expected scores (*y*‐axis) and thetas (*x*‐axis). The ‘all items graphs’ show the impact of DIF on the expected score when all items are combined, whereas the ‘DIF items graphs’ show the impact of DIF when only DIF items are considered. The graphs show that DIF had a minimal impact on the expected score when all items are administered.

**Table 3:** McFadden's pseudo R^2^ and IRT parameters for items displaying DIF

Item	Item content†	McFadden's pseudo R^2^ uniform DIF	*p*‐value uniform DIF	McFadden's pseudo R^2^ non‐uniform DIF	*p*‐value non‐uniform DIF	Discrimination α	Threshold β1	Threshold β2
Items with DIF for age								
VA4	Alternating visual attention	0.022	0.003	0.000	0.81	<5 years: 1.62 ≥5 years: 1.49	<5 years: 0.61 ≥5 years: 0.01	<5 years: 2.02 ≥5 years: 1.69
SR3	Playing with children without a visual impairment	0.016	0.009	0.002	0.39	<5 years: 1.67 ≥5 years: 2.00	<5 years: 0.41 ≥5 years: −0.06	<5 years: 1.75 ≥5 years: 1.20
SE3	(Un)dressing independently	0.032	<0.001	0.000	0.90	<5 years: 2.15 ≥5 years: 1.82	<5 years: −0.62 ≥5 years: −0.06	<5 years: 0.45 ≥5 years: 1.02
Items with DIF for gender								
SR4	Participating in group activities	0.022	0.002	0.007	0.087	Boys: 3.12 Girls: 2.21	Boys: −0.75 Girls: −0.55	Boys: 0.24 Girls: 0.73
MF1	Cutting and pasting	0.023	<0.001	0.000	1.0	Boys: 2.14 Girls: 2.03	Boys: −0.70 Girls: −0.24	Boys: 0.37 Girls: 0.79
SE3	(Un)dressing independently	0.018	0.003	0.005	0.12	Boys: 2.21 Girls: 3.00	Boys: −0.38 Girls: 0.04	Boys: 0.59 Girls: 0.72
Items with DIF for level of VI								
AT1	Recognizing facial expressions	0.057	<0.001	0.000	0.81	No‐mild VI: 0.87 Mod VI‐blind: 0.86	No‐mild VI: 0.70 Mod VI‐blind: −0.75	No‐mild VI: 3.17 Mod VI‐blind: 1.30
MF2	Climbing and clambering	0.012	0.031	0.016	0.015	No‐mild VI: 1.19 Mod VI‐blind: 2.17	No‐mild VI: −0.53 Mod VI‐blind: 0.10	No‐mild VI: 1.75 Mod VI‐blind: 1.37

AT, attachment; MF, motor functioning; SE, self‐reliance; SR, social relationships; VA: visual attention.

^†^Not an official (forward‐backward) translation.

### Final GRM

**Figure 2:**
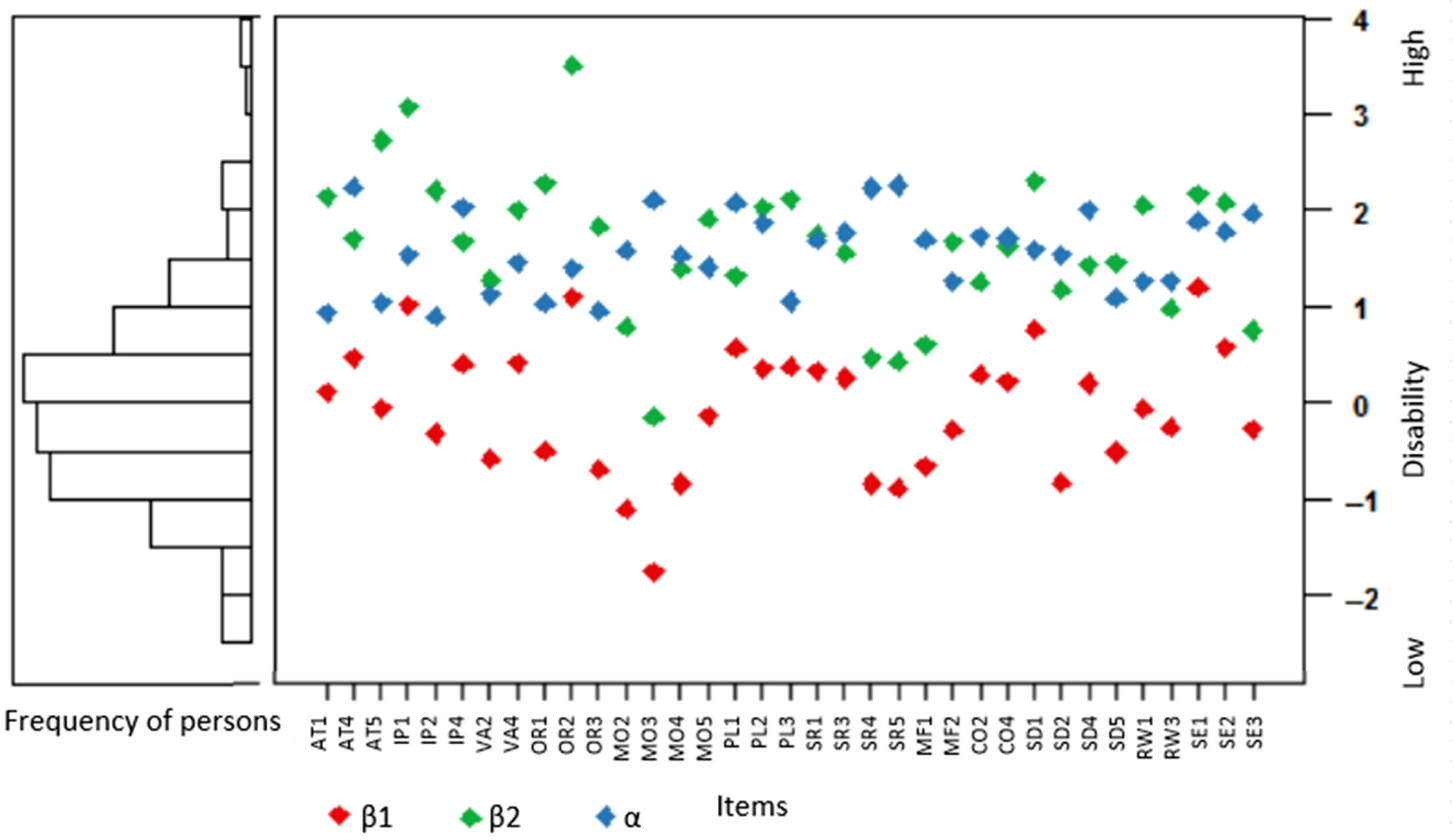
Item‐person map of the 35‐item PAI‐CY 3‐6.

For the 35 items, the full GRM with variable discrimination parameters outperformed the constrained model with equal discrimination parameters (LRT = 132.46, df = 34, *p* < 0.001). The SRMR fit index was adequate (0.077), whereas the other fit indices were reaching the preferred values (RMSEA = 0.087, TLI = 0.933 and CFI = 0.937). GRM item parameters, information and fit statistics for the PAI‐CY 3‐6 are displayed in *Table* *4*. Item discrimination ranged from 0.90 for item IP2 to 2.26 for item SR5, and item threshold parameters ranged from −1.76 to 3.50. Item information ranged from 1.46 to 4.01, and total information was 92.68. Although some items provided little information, further item removal was considered unfavorable for reasons of content validity, or because of their location on the latent trait. Only one item (OR2) showed misfit to the GRM at the *p* < 0.01 level (*p* = 0.005). The peaks of the second response category in the CRCs of three items (PL1, CO2, and RW3) were not as distinctive as they should. Considering all results, it was decided not to delete these four items. The item‐person map in *Figure* *2* shows that the items are distributed almost entirely over the latent trait, but majority of children have thetas at the lower side of the disability range (i.e. theta < 0), whereas the majority of items are at the higher end of the disability range (theta > 0). There are almost no respondents with thetas around 3.

**Table 4:** GRM item parameter estimates, item fit and parameters for test‐retest reliability for the 35‐item PAI‐CY 3‐6

Item	Item content†	Discrimination α	Threshold β1	Threshold β2	Item information	Χ^2^	*p*‐value	Agreement %	Weighted kappa
AT1	Recognizing facial expressions	0.93	0.10	2.14	1.46	12.4	0.50	74.1	0.72
AT4	Imitating actions or behavior	2.42	0.46	1.70	3.90	7.3	0.20	81.1	0.75
AT5	Exploring the environment independently	1.05	−0.06	2.72	1.84	17.1	0.072	66.0	0.51
IP1	Reacting to visual stimuli	1.54	1.01	3.07	2.76	4.8	0.31	75.9	0.43
IP2	Reacting to (sudden) sounds	0.90	−0.32	2.20	1.47	15.1	0.30	67.7	0.61
IP4	Executing tasks	2.03	0.40	1.67	3.45	6.5	0.37	75.0	0.69
VA2	Looking at something for a longer time	1.13	−0.59	1.27	1.82	13.8	0.46	64.7	0.67
VA4	Alternating visual attention	1.45	0.41	2.00	2.41	14.5	0.11	72.0	0.63
OR1	Orienting in a room	1.03	−0.50	2.28	1.81	16.1	0.19	63.6	0.55
OR2	Exploring the environment by touch	1.40	1.09	3.50	2.55	14.9	0.005	72.7	0.43
OR3	Finding toys in a closet or toy box	0.95	−0.70	1.83	1.59	10.4	0.73	69.2	0.65
MO2	Cycling	1.58	−1.11	0.79	2.80	11.2	0.43	80.3	0.82
MO3	Participating in traffic	2.10	−1.76	−0.15	3.83	5.3	0.50	82.2	0.81
MO4	Participating in physical activity classes	1.52	−0.84	1.39	2.78	16.7	0.082	79.0	0.76
MO5	Playing outside	1.41	−0.13	1.91	2.47	10.0	0.44	75.1	0.70
PL1	Playing imaginable games	2.08	0.57	1.32	3.08	8.3	0.22	85.6	0.85
PL2	Manipulating toys	1.86	0.35	2.03	3.33	5.9	0.43	80.1	0.76
PL3	Entertaining alone	1.05	0.37	2.11	1.63	20.6	0.056	84.7	0.85
SR1	Making contact with other children	1.69	0.33	1.74	2.82	8.8	0.36	75.7	0.71
SR3	Playing with children without a visual impairment	1.76	0.25	1.55	2.91	7.3	0.60	69.5	0.67
SR4	Participating in group activities	2.23	−0.84	0.47	3.93	21.3	0.012	69.4	0.69
SR5	Keeping up with other children while playing	2.26	−0.89	0.43	4.01	5.4	0.80	66.9	0.70
MF1	Cutting and pasting	1.69	−0.66	0.60	2.74	25.2	0.022	77.1	0.81
MF2	Climbing and clambering	1.27	−0.28	1.68	2.14	10.3	0.50	79.9	0.79
CO2	Sharing experiences	1.73	0.29	1.25	2.60	6.9	0.65	80.0	0.81
CO4	Asking for help	1.71	0.22	1.63	2.87	7.9	0.44	80.2	0.76
SD1	Finding the way in school	1.59	0.76	2.31	2.69	11.3	0.046	76.8	0.52
SD2	Maintaining overview in the classroom	1.54	−0.83	1.17	2.75	13.1	0.29	71.1	0.64
SD4	Getting insight in concepts	2.00	0.20	1.42	3.37	1.9	0.96	81.1	0.81
SD5	Maintaining enough energy after school for fun activities	1.08	−0.51	1.45	1.75	17.8	0.22	67.2	0.66
RW1	Recognizing pictures	1.26	−0.06	2.05	2.17	9.1	0.52	69.7	0.61
RW3	Recognizing letters	1.27	−0.25	0.98	1.87	15.1	0.37	75.0	0.76
SE1	Drinking independently	1.88	1.20	2.17	2.90	0.7	0.72	89.8	0.83
SE2	Eating independently	1.78	0.57	2.07	3.05	12.8	0.076	82.4	0.78
SE3	(Un)dressing independently	1.97	−0.28	0.75	3.14	6.1	0.81	79.9	0.84

AT, attachment; CO, communication; IP, incentive processing; MF, motor functioning; MO, mobility; OR, orientation; PL, play; RW, reading and writing; SD, school/daycare; SE, self‐reliance; SR, social relationships; VA, visual attention.

^†^Not an official (forward‐backward) translation.

### Known‐group validity, concurrent validity, test‐retest reliability

Independent samples t‐tests showed that those with comorbidity had significantly higher thetas (representing higher disability) than those without comorbidity (*p* < 0.001), indicating the PAI‐CY 3‐6 was able to discriminate between these groups. No significant differences were found between any other groups. However, after correcting for other variables in multiple linear regression, a significant association between thetas and severity of visual impairment (*p* = 0.004) and comorbidity (*p* < 0.001) was found, in which more severe VI was associated with higher thetas indicating higher disability, as was the presence of comorbidity. Correlations between sum scores of the PAI‐CY 3‐6 and sum scores of the CASP scales were all above the expected minimum value and significant at the *p* < 0.01 level demonstrating concurrent validity of the PAI‐CY 3‐6: home participation *r* = −0.78, community participation *r* = −0.74, school participation *r* = −0.70, home and community living activities *r* = −0.70, and total score *r* = −0.82. All items had satisfactory test‐retest reliability. Kappa values were moderate for five items, good for 22 items and very good for 11 items. Furthermore, 14 items showed moderate agreement and 24 items showed good agreement (*Table* *4*). Differences in IRT parameters for the test and retest were generally small. Mean difference in threshold β1 was 0.11 ± 0.08 (range 0.02–0.33), in threshold β2 0.18 ± 0.13 (range 0.01–0.47), and in discrimination α 0.25 ± 0.13 (range 0.02–0.71).

### Evaluation of the PAI‐CY 3‐6

The evaluation form was completed by 234 parents. Various aspects of the PAI‐CY 3‐6 were perceived neutral to positive by over 85% of the respondents (*Table* *5*). Mean self‐reported completion time of the PAI‐CY 3‐6 with 52 items, including questions on demographic and clinical characteristics, was 22 ± 11 (range 4–65, median 20) min (two persons were excluded because of implausible values, i.e. 0 and 10 000 min). Thirty‐nine parents indicated that they missed certain topics or questions in the PAI‐CY 3‐6, but upon inspection none of the suggestions provided by parents were mentioned by more than two respondents. Furthermore, 44% of the suggestions were not related to participation or activities, but instead concerned more general questions related to the character of the child, background information about the child, and progression of the visual impairment. Three parents stated that questions were primarily aimed at younger children, whereas for the individual items parents sometimes commented that their child was either too young or too old. It was suggested to depend usage of the PAI‐CY 3‐6 on the school grade a child is in, instead of using the strict age‐criteria. Professionals of low vision rehabilitation centers suggested to include two new items in the mobility domain (walking and swimming lessons). With the three maintained items as suggested by professionals of low vision rehabilitation centers, this resulted in a final PAI‐CY 3‐6 with 40 items (although scoring is based on the 35 items).

**Table 5:** Evaluation of the PAI‐CY 3‐6 by parents (*n* = 234)

Meaningfulness PAI‐CY 3‐6 for insight in possibilities of rehabilitation, *n* (%)	
Meaningless	4 (1.7)
Not meaningful	13 (5.6)
Neutral	69 (29.5)
Meaningful	130 (55.6)
Very meaningful	18 (7.7)
Representation of commonly experienced challenges by the child in the PAI‐CY 3‐6, *n* (%)	
Bad	13 (5.6)
Moderate	16 (6.8)
Reasonable	72 (30.8)
Good	114 (48.7)
Very good	19 (8.1)
Difficulty choosing the appropriate response category for the child in the PAI‐CY 3‐6, *n* (%)	
Always/almost always	1 (0.4)
Often	6 (2.6)
Regularly	25 (10.7)
Sometimes	114 (48.7)
Never/almost never	88 (37.6)
Satisfaction with administration time of the PAI‐CY 3‐6, *n* (%)	
Very unsatisfied	3 (1.3)
Unsatisfied	3 (1.3)
Neutral	39 (16.7)
Satisfied	146 (62.4)
Very satisfied	43 (18.4)

## Discussion

In this study some psychometric properties of the PAI‐CY 3‐6, a proxy‐instrument to assess the participation needs of children aged 3–6 years with VI, were evaluated using an IRT model. The 35‐item instrument has psychometrically sound properties, and is relatively short and easy to complete. It comprises a unidimensional scale with high measurement precision, and the items are distributed over the entire latent trait, thereby targeting the full range of children aged 3–6 years with VI. The PAI‐CY 3‐6 has good concurrent validity, and the strong correlations with scales of the CASP provides evidence that the construct measured is indeed participation. Furthermore, the PAI‐CY 3‐6 was able to distinguish between level of VI after correcting for potential confounders, and test‐retest reliability was adequate.

Although many ophthalmological instruments have been validated with models from the Rasch‐family (e.g.^16^, ^17^, ^56^, ^57^), such as rating scale models, we applied the GRM, which is a cumulative probability model, to assess the psychometric properties of the PAI‐CY 3‐6. There are several advantages of using Rasch models, such as statistical sufficiency and straightforward interpretation of the output. However, Rasch models in general, and the rating scale model specifically, are often too restrictive.^40^, ^47^ Satisfactory model fit can often only be obtained after deleting relatively large numbers of items, compromising face and content validity. A less constrained model, such as the GRM, often provides a more accurate reflection of the data.^48^ Use of the GRM is also advocated by the PROMIS initiative,^55^ comprising a precise, flexible, and comprehensive measurement system of over 300 PROMs of global, physical, mental and social health for adults and children in the general population and those living with a chronic condition.^58^ Moreover, the cognitive processes involved in selecting a response option in a Likert scale also favors the GRM over Rasch models.^59^, ^60^, ^61^ Other advantages of using the GRM include the robustness to slight deviations from normality^62^, ^63^ and the possibility to investigate non‐uniform DIF and item information.

To our knowledge, only two instruments for use in children aged 3–6 years are currently available, of which only the CVFQ is made specifically for children with VI (the PedEyeQ is aimed at the evaluation of eye‐related concerns across the entire spectrum of childhood eye conditions).^19^, ^20^ Unlike the PAI‐CY 3‐6, the CVFQ measures vision‐related quality of life and has not been developed involving the target‐population or end‐users.^19^ The PAI‐CY 3‐6 has strong and unique content validity, because it was firmly grounded in a population of children aged 3–6 years with VI, whose parents and rehabilitation professionals have shaped its content.^2^ Care was taken to ensure that content validity was retained in the process of item deletion, amongst others by checking whether the rehabilitation needs parents expressed after each domain could still be identified by the remaining items. Furthermore, the experience of low vision rehabilitation professionals who are going to use the instrument at the diagnostic assessment procedure was taken into account. They often have years of experience with many different children, which makes their opinion highly valuable. Moreover, it is important that professionals are satisfied with the PAI‐CY 3–6 years in order to achieve successful implementation in future. Involvement of professionals in the validation process may result in better understanding of the relevance of the PAI‐CY 3‐6, and increased satisfaction with the questionnaire. This will ultimately contribute to successful implementation of the instrument in Dutch low vision rehabilitation care.^64^, ^65^ Their feedback has led to the addition of two new items and the maintenance of three original items, although these are not included in the scoring. When the PAI‐CY 3‐6 is used as an outcome measure in research, it is advised to use the 35‐item instrument.

Similar to previous validation studies of instruments intended for pediatric ophthalmology,^17^, ^18^ the PAI‐CY 3‐6 seemed better targeted to children with high disability scores. This suggests that it may be particularly useful for the intended target‐population of low vision rehabilitation centers in the Netherlands, i.e. those with a visual acuity logMAR > 0.52 or a visual field of <30 degrees, or a clear rehabilitation need that cannot be solved within regular ophthalmic care.^66^ In this study, the match between respondents’ thetas and item thresholds was suboptimal, with more participants being on the lower side of the disability continuum, whereas items were overrepresented at the higher end. This is likely to be caused by the relatively large share of participants having no or mild VI according to the WHO criteria (almost 50%).^28^ These participants might not have been eligible for care by low vision rehabilitation centers, but instead might only have received diagnostic tests, and be therefore registered clients and as such invited to participate in this study. On the other hand, it might be that some of these children had cerebral visual impairment (CVI), in which visual acuity and visual field is often not affected, but visual function is impaired because of brain damage.^67^, ^68^ Moreover, measuring visual acuity and visual field in children this young age can be difficult, and often diagnoses are not yet established, and therefore, no definite explanations for the mismatch between participants and items can be made. However, it is reassuring that items are largely located over the entire disability trait. The high density of items at the higher end of the disability continuum might indicate that the PAI‐CY 3‐6 is particularly useful for capturing changes in participation over time for those children with high disability scores who are offered an intervention. Nevertheless, evaluating the responsiveness of the PAI‐CY 3‐6 should be subject to further study.

Seven items were found to show DIF, and these were not all in the expected direction. One of the two items that showed DIF for level of VI had uniform DIF, and as expected those with more severe VI had greater difficulty than those with less severe VI. However, two of the three items that showed DIF for age were more difficult for older children than for younger children. The reason for this is unclear, because the content of the items, i.e. ‘alternating visual attention’ and ‘playing with children without a visual impairment’, were not suggestive for older children to have greater difficulty endorsing these items. Moreover, all items showing DIF for gender were more difficult to endorse for boys than they were for girls. Two of the three items involved fine motor skills, i.e. ‘cutting and pasting’ and ‘(un)dressing independently’. Although contradicted by studies not finding any differences,^69^, ^70^, ^71^, ^72^ some studies have suggested that fine motor skills develop earlier in girls than in boys,^73^, ^74^ which might explain the DIF for gender found in this study. Upon inspection, DIF for gender did not seem to be confounded by differences in level of VI, presence of comorbidity or differences related to age. We chose not to delete the items displaying DIF, because DIF had minimal impact on the total score when all items are administered. However, if in future a computer adaptive test or short form is developed, it is important to reconsider the items displaying DIF and omit these items if possible.

Further development of the PAI‐CY 3‐6 is warranted, including evaluation of the newly added items and investigation of responsiveness over time, which will further confirm its validity and reliability. However, the psychometric properties demonstrated thus far are adequate for formal implementation into routine low vision rehabilitation practice. A large majority of the parents were satisfied about several aspects of the PAI‐CY 3‐6, although suggestions for further improvement were also made. Because suggestions were mentioned by only two respondents at most, we have not incorporated these. However, care must be taken that all rehabilitation needs are identified with the PAI‐CY 3‐6, and whether the response options ‘not difficult’, ‘little difficult’ and ‘very difficult/impossible’ are sufficient. In the PAI for young adults, the answer option ‘difficult’ was added because respondents suggested that the gap between ‘little difficult’ and ‘very difficult/impossible’ was too wide.^75^ This was also mentioned in this study, but only by two participants. In addition, respondents might have opted for ‘not applicable’ when they instead could have opted for ‘impossible’, causing data attrition. This phenomenon likely has happened in similar studies,^16^ and therefore clear instructions on when to opt for ‘not applicable’ should be provided (e.g. when an activity is not relevant because of the age of the child). Furthermore, instead of using the strict age‐criteria for use of the PAI‐CY 3‐6, the school grade or developmental age of the child should be considered. When a child starts to learn reading and writing (in grade 3 in the Netherlands, usually around the age of 6–7 years), the PAI‐CY 7–12 might be more appropriate. With the planned implementation of the improved instrument, acceptability and feasibility for parents who are referred to low vision rehabilitation centers and for professionals working with it can be further assessed. In addition, it will enable us to test the instrument in the rehabilitation context for which it was designed.

In conclusion, this study found that the PAI‐CY 3‐6 (the Dutch instrument is available upon request from the corresponding author) has sound psychometric properties to assess the participation needs of children aged 3–6 years with VI by means of parent or caregiver proxy‐reports. It is a novel instrument to assess participation and activities in this young population. The questionnaire is relatively short and easy to complete, and can now be considered for implementation in routine low vision rehabilitation care, where it can be further optimized and its acceptability and feasibility can be examined. It can be used complementary to objective clinical measures, such as visual acuity and visual field, and other instruments that provide background information on the child and its family, to assess the rehabilitation needs from the perspective of the child and its parents. This will likely positively influence referral to rehabilitation programs, and lead to more personalized and better quality health care.

## Conflict of interest

The authors report no conflicts of interest and have no proprietary interest in any of the materials mentioned in this article.
